# Cigarette smoking, nicotine dependence, and motivation to quit smoking in South African male psychiatric inpatients

**DOI:** 10.1186/s12888-016-1123-z

**Published:** 2016-11-16

**Authors:** Jean-Louis Du Plooy, Muiruri Macharia, Chris Verster

**Affiliations:** Department of Psychiatry, Faculty of Medicine and Health Sciences, Stellenbosch University, Cape Town, South Africa

**Keywords:** Cigarette smoking, Nicotine dependence, Smoking cessation, South Africa, Male psychiatric patients

## Abstract

**Background:**

Smoking is the leading cause of preventable death worldwide and the prevalence is particularly high among psychiatric patients but recent international studies demonstrated that psychiatric patients are able and motivated to quit. The aim of this study was to evaluate cigarette smoking, nicotine dependence, and motivation for smoking cessation in male psychiatric inpatients in a sample of South African acute-care male psychiatric inpatients.

**Methods:**

All inpatients admitted during a 2-month period (April to May 2016) to the Stikland Hospital Acute Male Admissions Unit in Cape Town, Western Cape, were included. Subjects completed a survey including a set of tests: Global Adult Tobacco Survey (GATS), the Fagerström Test for Nicotine Dependence (FTND), and the Decisional Balance for Cigarette Smoking (DBCS) (6-item version). Demographic data were obtained from patients’ clinical charts.

**Results:**

Among the 160 new inpatients, 72.5% (*n* = 116) completed the survey. Of the 116 participants, 91.4% (*n* = 106) were current smokers of whom 82% (*n* = 87) smoked daily and 55.6% (*n* = 59) were identified as having high nicotine dependence (FTND ≥ 6). Although a large majority (71.7%; *n* = 76) of current smokers expressed positive perceptions regarding smoking, a notable proportion (59.4%; *n* = 63) still attempted to quit the habit in the preceding 12 months and daily smokers were less likely to quit. However, only a minor proportion of all current (43.4%; *n* = 46) and specifically daily (40.2%; *n* = 35) smokers were advised on smoking cessation by a health worker.

**Conclusion:**

This study confirms that, similar to populations elsewhere, rates of cigarette smoking among psychiatric inpatients in South Africa is exceedingly high. While patients are motivated to quit smoking, few were provided with the necessary advice. Our findings provide further support for the integration of smoking cessation support in mental health care.

## Background

Smoking is the leading cause of preventable death, associated with about 6 million deaths worldwide each year, with many of these deaths occurring prematurely [1]. People with serious mental illness die an average of 25 years earlier than the rest of the population [1, 2] and a large proportion of this health disparity can be attributed to smoking-related causes [3]. Mentally ill persons are twice as likely to smoke, to smoke more heavily and to be more nicotine-dependent than the general population [4]. Smoking rates are even higher in individuals with severe mental illnesses such as schizophrenia and bipolar disorder, of whom between 30% and 70% respectively, consume more than 20 cigarettes per day [5].

Besides a reduced life expectancy and other health-related issues, smoking has a profound social impact. One major impact is financial burden as people with mental illness spend a disproportionate amount of their income on cigarettes while struggling to afford food or other essential daily needs [6, 7]. Moreover, smoking adversely affects health and life-long cigarette smokers have a higher prevalence of conditions such as malignancy, cardiovascular disease and chronic obstructive pulmonary disease [8], increasing chronic morbidity and mortality. The relationship between smoking and mental illness seems to be bi-directional, with each contributing to the other. Thus the presence of smoking relates to higher incidences of poor mental health, while those with poor mental health have a greater likelihood of being smokers [9]. In that study, women who smoked were up to 1.6 times more likely to have poor mental health while those with poor mental health were more than twice (odds: 2.1) as likely to smoke. A Dutch study has shown earlier ages of onset for depression and anxiety disorders in those who started smoking at an earlier age after controlling for relevant confounders [10]. In contrast, smoking cessation has been associated with reduced depression, anxiety and stress, with improved quality of life even in the absence of mental illness [11]. Smoking also increases metabolism of various antipsychotic agents, thus resulting in lower serum concentrations of antipsychotic medication and other medications metabolized by the cytochrome P450 system [12]. This may result in sub-therapeutic levels of treatment, with compromised benefit to the patient and greater potential for acute relapses of psychotic symptoms in e.g. schizophrenia [12]. Many of the smoking-induced changes are dose–response related and reversible after cessation.

There is no consensus whether people with mental health problems are less likely than those unaffected to want, or be able, to quit smoking. One theory is that mentally ill people belong to a category of so-called ‘hardcore’ smokers defined as daily, long-term smokers who, despite extensive knowledge of the health hazards of smoking and substantial social pressure to quit, are completely unwilling or unable to do so [13]. On the one hand, there is indeed a moderate amount of evidence suggesting that mentally ill persons find it difficult to quit smoking, which may be explained by a number of factors. To some of the patients, smoking offers a sense of control of symptoms and freedom in a setting of limited control [14]. Smoking also gives the patients sense of identity and encourages friendship, in an otherwise socially isolated and often stigmatized group [14]. In addition, inpatient environments may promote smoking behavior through boredom and peer pressure [14]. On the other hand, there is some evidence that mentally ill persons are as motivated to quit, and benefit from smoking cessation treatments and interventions as much as the general population [15]. However, these patients have in the past been less likely to receive such interventions [16] stemming from untested perceptions among health care workers that, because of multiple impairments, quitting smoking may present particular challenges for them [17].

Although global and South African prevalence of smoking has decreased in the general population as a result of anti-smoking regulations and extensive public campaigns [18–20], rates have declined much less, if at all, in people with mental disorders [21].

Data related to smoking cessation in South Africa, particularly in mentally ill patients, is conspicuously lacking and thus the aim of this study was to evaluate the rate of smoking, nicotine dependence, and motivation to quit the habit in a sample of South African acute-care male psychiatric inpatients. The aligning of local legislation with the World Health Organization Framework Convention on Tobacco Control [22] has made smoking in public spaces increasingly difficult. The most recent Tobacco Control Amendment Act (63 of 2008) prohibits tobacco smoking in a public place, or within a prescribed distance from a window of, ventilation inlet of, doorway to or entrance into a public place [23–26]. Most health care facilities in South Africa are not yet compliant, but if applied, this law positions hospitalization as an excellent opportunity for mental health workers to play a guiding role in reducing rates of smoking within this key population.

## Methods

### Settings and subjects

The study sample was drawn from inpatients at the acute-male admissions unit of Stikland Hospital. The hospital is a state facility and the only specialist psychiatric facility providing acute psychiatric inpatient services to the mixed urban and rural community of approximately 1.5 million people in the northern suburbs of Cape Town. Genders are separated in the unit and there are 80 beds for male patients. With emphasis on acutely psychotic patients, consecutive inpatient admissions during the 2-month period of April- May 2016 were screened and approached to participate if they were 18–59 years, clinically stable, willing to give consent, literate enough to complete the questionnaire and able speak either of the two languages used in the study. All participants gave written informed consent and participated in the study according to locally and internationally accepted ethical guidelines. The study was approved by the Health Research Ethics Committee of the Stellenbosch University in Cape Town, South Africa (HREC# S15/10/230).

### Data collection

Data were obtained from medical records and via a structured interview that included completion of a guided, 15-min questionnaire that was administered by a psychiatry registrar familiar with the study tools. The records review and interview provided socio-demographic and co-morbidity data including age, marital status, highest level of education, employment and the working diagnosis at the time of assessment. For the purposes of this study, working diagnosis was limited to the primary psychiatric diagnosis.

The 3-section questionnaire consisted of a modified version of the Global Adult Tobacco Survey (GATS), the Fagerström Test for Nicotine Dependence (FTND), and the Decisional Balance for Cigarette Smoking (DBCS) (6-item version). The frequency of present and past smoking (daily, less than daily, not at all) was assessed using a subset of key questions from the “Tobacco Questions for Surveys”, a component of the GATS [27]. Relevant to the population surveyed, a specific distinction was made to number of both manufactured and hand-rolled cigarettes smoked. Further questions aimed to establish whether, in the preceding 12 months, participants had attempted to quit smoking or been advised by a health professional about smoking cessation. To assess nicotine dependence, we used the 6-item FTND which has a graded scale (0–10) corresponding to the degree of nicotine dependence [28]. The FTND is based on DSM-IV criteria for nicotine dependence and a score of ≥6 is widely used as the cut-off. The 6-item DBCS [29] was used to assess the pros and cons of smoking, with higher scores indicating greater importance of pros or cons for the individual.

### Data analysis

IBM SPSS version 22 [30] was used to analyze the data and *p* value <0.05 was set as the level of statistical significance. Categorical variables were described using count and percentages, while continuous variables were described using means and standard deviations. Student’s *t*-tests and Chi squares tests were used to compare quantitative and qualitative traits respectively, between occasional and daily smokers.

## Results

### Demographics

A total of 160 patients were admitted to the study unit during the survey period of whom 44 (27.5%) were excluded on the basis of psychiatric instability (*n* = 11; 6.9%), discharge prior to interview (*n* = 11; 6.9%), unavailability during interviews (*n* = 1; 0.6%), declined consent (*n* = 4; 2.5%) and inability to write (*n* = 6; 3.8%) or speak either of the two languages represented among the staff (*n* = 11; 6.9%). Thus the final analytic sample comprised 116 (72.5%) for whom the socio-demographic characteristics are presented in Table 1. Their age ranged from 18 to 59 with a mean of 30.3 ± 9.5 years and the majority was single (*n* = 103; 88.8%), unemployed (*n* = 99; 85.3%) and educated to secondary level (*n* = 83; 71.6%). The most common primary diagnoses were schizophrenia (*n* = 54; 46.6%), substance induced psychotic disorder (SIPD) (*n* = 26; 22.4%) and bipolar disorder (*n* = 14; 12.1%) while psychotic disorder due to another medical condition (AMC) (*n* = 3; 2.6%) was the least encountered.

**Table 1:** Participants’ demographic profile

Characteristic	Frequency
Age, mean (SD)	30.3 (9.5)
Marital status, *n* (%)	
Single	103 (88.8)
Married	7 (6.0)
Divorced/widowed	6 (5.1)
Unemployed, *n* (%)	99 (85.3)
Highest education, *n* (%)	
Secondary level	83 (71.6)
Primary level	20 (17.2)
Other/unknown	13 (11.2)
Diagnosis, *n* (%)	
Schizophrenia	54 (46.6)
SIPD	26 (22.4)
Bipolar Disorder	14 (12.1)
Schizoaffective	12 (10.3)
Schizophreniform	7 (6.0)
Psychosis due to AMC	3 (2.6)
Smoking status, n (%)	
Daily smoker	87 (75.0)
Less than daily	19 (16.4)
Non-smoker	10 (8.6)

### Smoking and nicotine dependence

As shown in Table 2, a vast majority of the participants (*n* = 106; 91.4%) were identified as current smokers of whom 87 (82%) indicated to smoking daily and the rest (*n* = 19; 18%) less-than-daily. On a daily basis, the largest proportion of the smokers consumed ≤10 (*n* = 46; 43.4%) and 11–20 (*n* = 42; 39.6%) cigarettes while 17% (*n* = 18) smoked more than ≥21cigarettes. The majority (*n* = 59; 55.7%) were classified as having high or very high nicotine dependence as assessed by the FTND.

**Table 2:** Participants’ smoking related characteristics^a^

Variable	Overall (*n* = 106)	Occasional smoker (*n* = 19)	Daily smoker (*n* = 87)	*p*
Age years (SD)	30.1 (9.4)	27.7 (7.9)	30.7 (9.7)	0.21
Diagnosis				
Schizophrenia	49	10	39	
SIPD	25	2	23	
Bipolar	11	1	10	
Schizoaffective	12	2	10	0.180
Schizophreniform	6	3	3	
Psychosis due to AMC	3	1	2	
Cigarettes smoked per day				
≤ 10	46 (43.4)	11 (57.9)	35 (40.2)	
11–20	42 (39.6)	5 (26.3)	37 (42.5)	0.227
21–30	11 (10.4)	3 (15.8)	8 (9.2)	
≥ 31	7 (6.6)	0 (0.0%	7 (8.0)	
Nicotine dependence				
0–2 (Very low)	11 (10.4)	4 (21.1)	7 (8.0)	0.093
3–4 (Low)	18 (17)	4 (21.1)	14 (16.1)	
5 (Moderate)	18 (17)	3 (15.8)	15 (17.2)	
6–7 (High)	41 (38.7)	8 (42.1)	33 (37.9)	
8–10 (Very high)	18 (17)	0 (0.0)	18 (20.7)	
FTND score, mean (SD)	5.4 (2.2)	4.3 (2.4)	5.7 (2.1)	0.009
Cigarette would hate most to give up				
First one in morning	79 (74.5	12 (63.2)	67 (77.0)	0.248
Any other	27 (25.5)	7 (36.8)	20 (23.0)	
How soon after waking up do you smoke your first cigarette				
Within 5 min	5 (4.7)	0	5 (5.7)	0.528
5–30 min	32 (30.2)	5 (26.3)	27 (31.0)	
31–60 min	56 (52.8)	10 (52.6)	46 (52.9)	
Other	13 (12.3)	4 (21.1)	9 (10.3)	
Is it difficult to refrain from smoking in “no-smoking” places				
Yes	56 (52.8)	4 (21.1)	52 (59.8)	
No	50 (47.2)	15 (78.9)	35 (40.2)	0.002
Do you smoke even when ill bedridden most of the day				
Yes	55 (51.9)	6 (31.6)	49 (56.3)	0.075
No	51 (48.1)	13 (68.4)	38 (43.7)	
Smoke more frequently first hours after waking than rest of day				
Yes	65 (61.3)	8 (12.3)	57 (87.7)	0.071
No	41 (38.7)	11 (57.9)	30 (34.5)	
Perception on smoking				
Negative	25 (23.6)	6 (31.6)	19 (21.8)	
Positive	76 (71.7)	12 (63.2)	64 (73.6)	0.218
Neutral	5 (4.7)	1 (5.3)	4 (4.6)	
Attempted to quit in the past 12 months				
Yes	63 (59.4)	16 (84.2)	47 (54.0)	0.019
No	43 (40.6)	3 (15.8)	40 (46.0)	
Advised to quit smoking by health worker in past 12 months				
Yes	46 (43.4)	11 (57.9)	35 (40.2)	0.299
No	58 (54.7)	8 (42.1)	50 (57.5)	

^a^Results are presented as count (%) unless specified otherwise. *AMC* another medical condition

### Smokers’ perception about their smoking and motivation & advice to quit smoking

Among the current smokers, the perceived positives of smoking outweighed the perceived negatives for the majority (*n* = 76; 71.7%), were less in 25 (23.6%) and neutral in 5 (4.7%). Nonetheless, a majority of the current smokers (*n* = 63; 59.4%) indicated they had tried to quit smoking in the 12 months preceding the study and the proportion was significantly higher among the non-daily smokers (*n* = 16; 84.2%) compared to daily smokers (*n* = 47; 54.0%; *p* = 0.019). Only a minority (*n* = 46; 43.4%) of the smokers, however, had received advice to quit smoking from a health professional in the preceding year. Interestingly, a smaller proportion of daily smokers (*n* = 35; 40.2%) received this advice compared to the less frequent smokers (*n* = 11; 57.9%).

## Discussion

In this study aiming to examine the rate of, motivation and proffered advice to quit smoking in acutely ill psychiatric male patients, we had several important findings. First, an overwhelming majority of the patients (*n* = 106; 91%) were classified as active smokers, a large proportion (*n* = 87; 75%) smoking on a daily basis. This is nearly three times the rate (32%) reported among the local general male population [31]. Our findings are consistent with past research [3, 16] in demonstrating exceedingly high rates of smoking in psychiatric patients. The high rate in our sample may be partly explained by the high numbers of schizophrenia diagnoses given the known association between the two [32, 33].

Second, dependence on tobacco, as reflected by the FTND scale, appears to be high with almost 56% of smokers showing high or very high nicotine dependency compared to less than 20% reported in the general population [34]. The mean FTND score in our study (5.4) is also higher than in the general population but comparable to the 4.6 and 6.2 reported by Solty et al. [35] and De Leon et al. [36] respectively in psychiatric samples.

Third, a positive perception regarding smoking was predominant among the smokers suggesting that most may not be currently contemplating quitting. Thus our finding that a majority had attempted to quit the habit in the year preceding the study was somewhat contrary. The finding is, however, consistent with past research [35, 37] and adds to the evidence that, like the general population, patients with mental illnesses are motivated to quit. Given that provider counseling doubles the likelihood that a smoker will quit [38], clinicians should therefore utilize the increasingly smoking-restricted environments provided by psychiatric hospitals to motivate these patients towards cessation. Yet, smoking cessation counseling appears to be a low priority even in the behavioral healthcare setting [39–41].

Consistent with the above observation, our fourth key finding was that although a majority of the smokers made efforts to quit, only a minor proportion received advice on smoking cessation from any health worker. This important finding is not unique to our study. Studies have repeatedly shown that patients with severe mental illnesses were less likely to receive advice and interventions to aid in smoking cessation [42–44]. Solty et al. [35], for example, reported much lower rates (36.2%) of patients provided with smoking cessation advice than found in our study. The finding could explain, at least partly, why declines in smoking prevalence observed in the general population have not been replicated in psychiatric populations [16].

This study has some limitations. The sample was restricted to male acute inpatient services at a single setting thus limiting the generalizability of the findings to other subpopulations e.g. females and outpatient. Given the nature of the sample, reliability of the responses is unknown although we endeavored to exclude clinically unstable subjects. The study was also limited by an emphasis on primary psychiatric diagnoses yet comorbidity, a common phenomenon in psychiatric patients, may substantially impact on various aspects related to smoking including prevalence, nicotine dependence and thoughts of quitting [35, 45]. Lastly, the sample size was a restriction on the performance of more interrogative analyses involving, for example, the relationships between psychiatric diagnoses and smoking/cessation. A longer observation time would certainly have yielded a larger sample size, but owing to logistical and institutional restrictions, data collection was limited to two months.

## Conclusions

In summary, our findings confirm that, similar to populations elsewhere, rates of cigarette smoking and tobacco dependence among South African psychiatric inpatients is exceedingly high. While many of these patients are motivated to quit and have made recent efforts in that regard, information that can support them in this endeavor seems inadequate as few were provided with pertinent advice echoing concerns that clinicians underutilize their considerable influence in helping patients adopt the healthier lifestyles. Given the detrimental impact that smoking has on health, therapeutic intervention and cessation efforts should be an integral part of available mental health care. As such, clinicians should utilize institutional smoking restrictions, similar to policies recently implemented at Stikland Hospital, to motivate psychiatric patients towards quitting and reducing smoking prevalence among these patients as has been achieved in the general population. Ideally, these efforts should extend beyond the inpatient setting given the increasing shift towards outpatient and community based mental health care.

## Abbreviations

AMC: Another medical condition
DBCS: Decisional balance for cigarette smoking
DSM-IV: Diagnostic and statistical manual of mental disorders
FTND: Fagerström test for nicotine dependence
GATS: Global adult tobacco survey
IBM SPSS: International business machines - statistical package for social sciences
SIPD: Substance induced psychotic disorder
